# Quercetin Antagonizes Glucose Fluctuation Induced Renal Injury by Inhibiting Aerobic Glycolysis via HIF-1α/miR-210/ISCU/FeS Pathway

**DOI:** 10.3389/fmed.2021.656086

**Published:** 2021-03-04

**Authors:** Wei-long Xu, Su Liu, Nan Li, Li-fang Ye, Min Zha, Chang-yin Li, Yue Zhao, Qiang Pu, Jin-jing Bao, Xing-jie Chen, Jiang-yi Yu, Ying-hao Pei

**Affiliations:** ^1^Department of Endocrinology, Jiangsu Province Hospital of Chinese Medicine, Affiliated Hospital of Nanjing University of Traditional Medicine, Nanjing, China; ^2^Department of Clinical Pharmacology, Jiangsu Province Hospital of Chinese Medicine, Affiliated Hospital of Nanjing University of Traditional Medicine, Nanjing, China; ^3^Department of Endocrinology, Rugao Hospital of Traditional Chinese Medicine, Nantong, China; ^4^Department of Intensive Care Unit, Jiangsu Province Hospital of Chinese Medicine, Affiliated Hospital of Nanjing University of Traditional Medicine, Nanjing, China

**Keywords:** glucose fluctuation, quercetin, renal injury, aerobic glycolysis, HIF-1α/miR-210/ISCU/FeS axis

## Abstract

**Background and Objective:** Glucose fluctuation (GF) has been reported to induce renal injury and diabetic nephropathy (DN). However, the mechanism still remains ambiguous. Mitochondrial energy metabolism, especially aerobic glycolysis, has been a hotspot of DN research for decades. The activation of HIF-1α/miR210/ISCU/FeS axis has provided a new explanation for aerobic glycolysis. Our previous studies indicated quercetin as a potential therapeutic drug for DN. This study aims to evaluate levels of aerobic glycolysis and repressive effect of quercetin via HIF-1α/miR210/ISCU/FeS axis in a cell model of GF.

**Methods:** The mouse glomerular mesangial cells (MCs) were exposed in high or oscillating glucose with or without quercetin treatment. Cell viability was measured by CCK8 assay. Aerobic glycolysis flux was evaluated by lactate acid, pH activity of PFK. Apoptosis level was confirmed by Annexin V-APC/7-AAD double staining and activity of caspase-3. TNF-α and IL-1β were used to evaluate inflammation levels.

**Results:** GF deteriorated inflammation damage and apoptosis injury in MCs, while quercetin could alleviate this GF-triggered cytotoxicity. GF intensified aerobic glycolysis in MCs and quercetin could inhibit this intensification in a dose-dependent manner. Quercetin prevented activities of two FeS-dependent metabolic enzymes, aconitase, and complex I, under GF injury in MCs. The mRNA expression and protein contents of HIF-1α were increased after GF exposure, and these could be alleviated by quercetin treatment. Knockdown of ISCU by siRNA and Up-regulating of miR-210 by mimic could weaken the effects of quercetin that maintained protein levels of ISCU1/2, improved cell viability, relieved inflammation injury, decreased apoptosis, and reduced aerobic glycolysis switch in MCs.

**Conclusion:** Quercetin antagonizes GF-induced renal injury by suppressing aerobic glycolysis via HIF-1α/miR-210/ISCU/FeS pathway in MCs cell model. Our findings contribute to a new insight into understanding the mechanism of GF-induced renal injury and protective effects of quercetin.

## Introduction

The incidence of diabetes mellitus (DM) has been increasing worldwide and become a major public health problem in China (1). Diabetic nephropathy (DN) is the most common chronic microvascular complication triggered by DM, which is the leading cause of end-stage renal disease (2). Glucose fluctuation (GF) has been reported to induce renal injury and be involved in the pathogenesis of DN. It was demonstrated that the short-term glucose variability was closely associated with decreased estimated glomerular filtration rate and an increased risk of CKD in DM patients (3). Other vitro studies indicated that unstable blood glucose had apoptosis-triggering effects on cells, including glomerular mesangial cell (4) and vascular endothelial cell (5). However, it still remains ambiguous under the mechanism between glucose variability and DN.

Mitochondrial energy metabolism has been a hotspot in DN research for decades, including aerobic glycolysis (the “Warburg effect”). Aerobic glycolysis flux, indicated by glucose uptake and lactate production, was increased in DN rats and increasing aerobic glycolysis could remarkably induce myofibroblasts activation and affected the number and function of podocytes (6, 7). The activation of HIF-1α/miR210/ISCU/FeS axis has provided a new explanation for aerobic glycolysis (8). MiR-210 is a response binding element of HIF-1α and represses its downstream molecules, iron-sulfur cluster assembly scaffold protein (ISCU), which mediates FeS assembly (9). Disturbance of FeS assembly contributes to the development of DN via inactivation of FeS-dependent enzymes, such as complex I (10). HIF-1α is also considered to play roles among GF (11), DN (12), and Warburg effect (13). Mitochondria are the major sites for regulating glucose metabolism of cells. In the condition of glucose intermittent, hypoxia-inducible factor 1α (HIF-1α) enhances its transcriptional activity triggered by dysfunction of mitochondria (11). Thus, it is meaningful to investigate whether HIF-1α/miR210/ISCU/FeS axis underlies aerobic glycolysis in conditions of GF induced renal injury.

Quercetin distributes in various fruits and vegetables and is one of bioflavonoid compounds of Abelmoschus plants, which has been reported as a potential therapeutic herb for the treatment of DN in our previous studies (14, 15) and other studies (16, 17). Quercetin shows diverse pharmacological effects, including anti-oxidation and anti-inflammatory (18, 19). It is reported that Quercetin could inhibited proliferation in high glucose–treated mouse glomerular mesangial cells (MCs) and in early DN mouse (20). Our previous study showed that quercetin presented protective effects against the initiation and progression of DN in diabetic mice by improving the renal accumulation of lipid bodies (21). A network pharmacology study demonstrated that Quercetin had a good binding on factors of inflammatory response, angiogenesis and oxidative stress reaction, which all involved in DN (22). Quercetin also held the ability to inhibit Warburg effect in many cells (23, 24) and downregulate HIF-1α to reduce renal oxidative stress apoptosis (25). However, the protective mechanism of quercetin against aerobic glycolysis in the GF induced renal injury has not been reported.

In the present study, we first evaluated levels of aerobic glycolysis and repressive effect of quercetin in a mouse MCs model of intermittent high glucose. Then, we elucidated the roles of HIF-1α/miR210/ISCU/FeS axis underlying these effects of quercetin.

## Materials and Methods

### Cell Culture and Treatment

The mouse glomerular MCs SV40 MES 13 was purchased from Cell Bank/Stem Cell bank (Shanghai Chinese Academy of Sciences). MCs were cultured in DMEM (5.56 mmol/L glucose) supplemented with 10% fetal bovine serum (FBS), antibiotics (100 U/ml penicillin and 100 mg/ml streptomycin), in a 5% CO_2_ humidified atmosphere at 37°C.

The cells were randomly divided into seven groups: normal glucose group (NG, 5.6 mmol/l glucose), high glucose group (HG, 50 mmol/l glucose), glucose fluctuation group (GF, alternated 5.6 mmol/l glucose and 50 mmol/l glucose every 8 h), GF+10 μmol/L quercetin group (GF+Q10, cells treated with GF in the presence of 10 μmol/L of quercetin), GF+20 μmol/L quercetin group (GF+Q20, cells treated with GF in the presence of 20 μmol/L of quercetin), GF+40 μmol/L quercetin group (GF+Q40, cells treated with GF in the presence of 40 μmol/L of quercetin), mannitol group (MG, 5.6 mM glucose plus 44.4 mM mannitol as an osmotic pressure control).

### Cell Transfection

The oligonucleotides were transfected into cells according to the manufacturer's instructions. Briefly, cells were seeded 24 h before transection to make sure 70–80% cell density. Then, Opti-MEM medium without antibiotics and serum was used to dilute the oligonucleotides (200nM ISCU1/2 siRNA, 100 nM miR210 mimic or inhibitor) and Lipofectamine 3000 transfection reagent (Invitrogen, USA). Subsequently, mix these two diluents. After 48 h of incubation in a 5% CO_2_ humidified atmosphere at 37°C, the transfection medium was replaced with fresh penicillin/streptomycin-free medium for 24 h before subsequent experiments. ISCU siRNA and control siRNA were purchased from Santa Cruz Biotechnology (Santa Cruz, CA). Both miR210 mimic and mimic control were purchased from Kaiji Biotech (Jiangsu, China).

### Cell Counting Kit-8 (CCK-8) Assay

The cells were plated in 96-well plates with 5 × 10^3^ cells per well. The cells were serum starved for 24 h after adherence, followed by different managements. Subsequently, cells were incubated in 10 μL CCK-8 (Dojindo Laboratories, Kumamoto, Japan) for 1 h. The optical density (OD) at 450 nm of each group was determined by a microplate reader (BioTek, USA). Mean OD value was calculated by triplicate repeats.

### Measurements of Lactate Acid and pH in Cell Supernatant

Lactate acid (lac) level was measured by using Lac Colorimetric/Fluorometric Assay Kit (Jiancheng Biotech., A019-2-1). The pH was measured with pH instrument (OHAUS STARTER 2C, USA) according to the manufacturer's instructions.

### PFK Activity Assay

PFK Activity Colorimetric Assay Kit was applied to evaluate the activity of phosphofructokinase (PFK) (Sigma-Aldrich, USA). Treated cells were mixed with PFK Assay Buffer and under cell lysis with Reaction Mix according to the manufacturer's instructions. Microplate reader was used to test the OD value of the mixtures per 30 s. One unit of PFK mediates 1.0 μM per minute of NADH generation. A standard line of NADH was built for PFK activity calculation. After normalization to the protein concentration, the PFK activity was showed as milliunits/mg of protein.

### Measurements of Aconitase and Complex I Activity Assays

Mitochondria were isolated using the Mitochondrial Isolation Kit for Cultured Cells from Abcam. The Complex I Enzyme Assay Kit (Abcam) and Aconitase Assay Kit (Sigma) were used to determine activity of Complex I and aconitase, respectively, according to the manufacturer's protocol. A Multi-Plate Reader was used to read the plate at a wavelength of 450 nm.

### Measurements of TNF-α and IL-1β

Levels of tumor necrosis factor alpha (TNF-α) and interleukin-1β (IL-β) in culture supernatants were quantified using commercially available ELISA kits conducted in accordance with the manufacturer's instructions.

### Annexin V-APC/7-AAD Double Staining

After treatment, MCs were harvested, washed and stained with Annexin-V APC/7-AAD cell apoptosis assay kit (Jiangsu Kaiji Biotech., KGA1024) according to the manufacturer's instructions. Four subpopulations were identified: normal cells (Annexin V-APC^−^/7-AAD^−^), necrotic cells (Annexin V-APC^−^/7-AAD^+^), early apoptotic (Annexin V-APC^+^/7-AAD^−^) and late apoptotic (Annexin V-APC^+^/7-AAD^+^). Apoptosis index was the total rates of early apoptotic and late apoptotic cells.

### Western Blotting Analysis

After measuring the protein concentrations, the cell lysates and subcellular fractionation were separated by SDS-PAGE and then transferred to PVDF membranes (Millpore, Billerica, MA, USA). Then, the membranes were blocked with 5% fat-free milk and incubated with anti-caspase3 (1:500, proteintech, 19677-1-ap), cleaved caspase3 (1:1,000, CST, 9664), anti-PKM2 (1:1,000, Beijing Boaosen Biotechnology, bs-0102M), anti-p-PKM2 (1:1,000, CST, 3827), HIF-1α (1:1,000, CST, 14179), and anti-ISCU (1:1,000, proteintech, 14812-1-AP), respectively. The bound antibodies were detected with 1:5,000 diluted goat-anti-rabbit IgG-HRP (Jiangsu Kaiji Biotech., KGAA35) and the bands were developed using an enhanced chemiluminescence ECL kit (Applygen Technologies). The relative levels of each protein to beta-actin were determined by the G: BOX ChemiXR5 imaging system.

### qRT-PCR Analysis

The first strand of cDNA was synthesized by M-MLV Reverse Transcriptase (Life Technologies). The RT-qPCR was performed as previously described (26). After normalizing with U6, the relative levels of target miRNAs were calculated by ΔΔCT method.

### Immunofluorescence Analysis

MCs cultured on glass coverslips were washed and fixed with 4% paraformaldehyde for 30 min. After three times of PBS washing, the cells were blocked with 10% ready-to-use goat serum for 20 min at room temperature, incubated with primary antibodies (1:100) at 37°C for 2 h, followed by incubation with a secondary antibody with FITC (1:100) at 37°C protected from light for 1 h. ISCU antibody was purchased from Wuhan Sanying Biotechnology (Wuhan, China, 14812-1-AP). Then, the cells were counterstained with DAPI at 37°C protected from light for 5 min. The coverslips were mounted onto glass slides. Fluorescence microscope was used to observe the expression of protein and take images of three high expression areas.

### Statistical Analysis

All experiments were repeated three times. Results were expressed as the mean ± standard deviation (SD). The difference among groups was analyzed by one-way ANOVA using SPSS 22.0 software. *P* < 0.05 was considered significantly different.

## Results

### Quercetin Protected Glomerular MCs From GF-Induced Inflammation and Apoptosis Injuries

**Figure 1:**
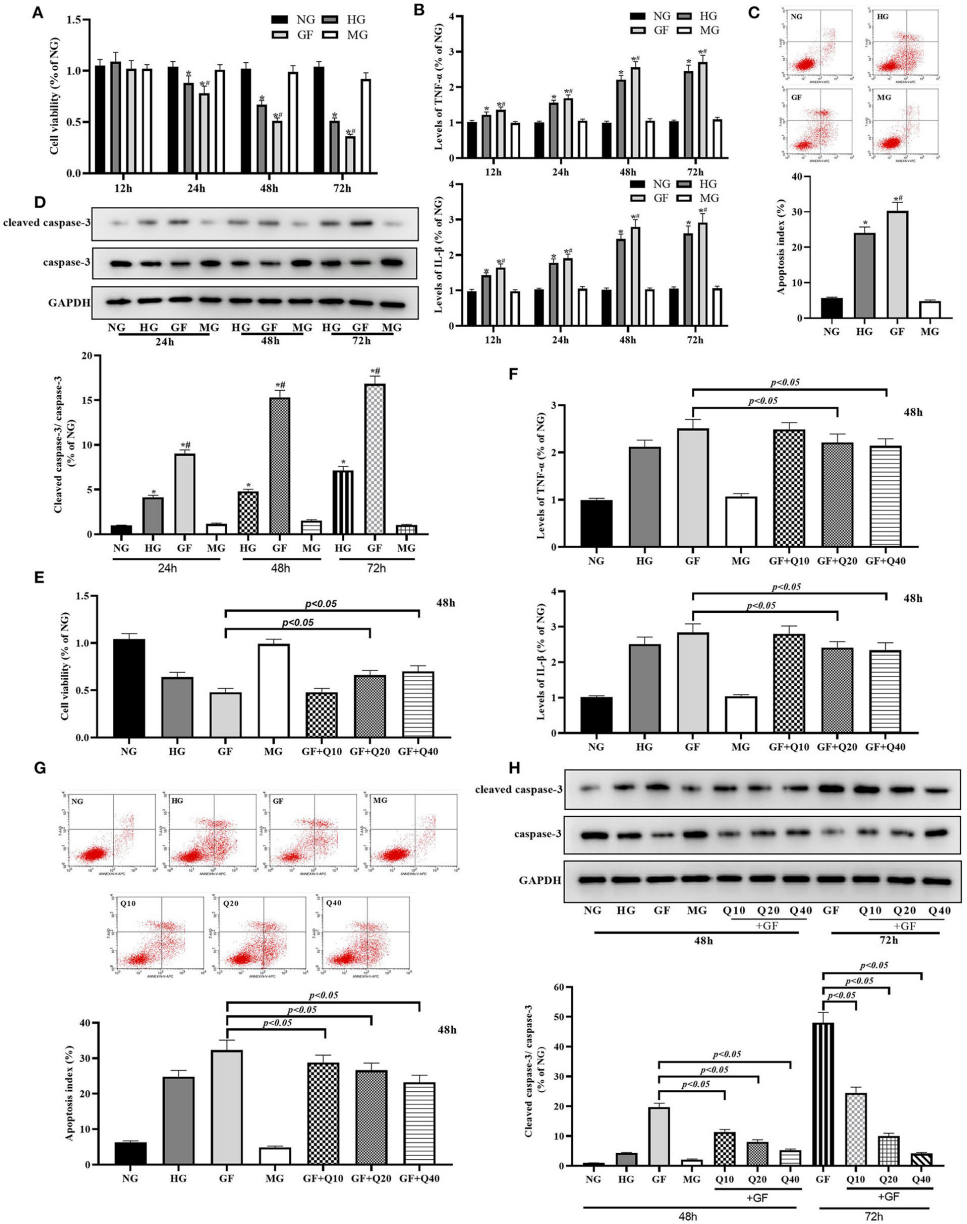
Quercetin protected glomerular MCs from GF-induced inflammation and apoptosis injuries. **(A)** Viability of MCs was tested by CCK8 at 24, 48, and 72 h. **(B)** Inflammation levels of TNFα and IL-1β at 24, 48, and 72 h. **(C)** Apoptosis index was detected by flow cytometry under different glucose at 48 h. **(D)** Rates of cleaved caspase-3/caspase-3 measured by WB analysis at 48 h. **(E,F)** Moderate (20 μmol/L) and high dose (40 μmol/L) of quercetin treatment presented protection effects on viability of MCs and levels of inflammation damage. **(G,H)** Effects of quercetin on apoptosis index and caspase-3 activity. The error bar reflects the S.E.M. of at least three independent experiments. *P < 0.05 vs. NG. ^#^*P* < 0.05 vs. HG.

We first explore the cytotoxicity of GF in MCs. As shown in Figure 1, the viability of MCs was significantly decreased in the FG group compared with the NG and HG groups at 24, 48, and 72 h, while no significant difference was found between NG and HG group (Figure 1A). In comparison of NG and HG, FG could significantly increase inflammation levels (TNFα and IL-1β) at 24, 48, and 72 h (Figure 1B). Flow cytometry and WB test showed that the apoptosis index and rate of cleaved caspase-3/caspase-3 were significantly increased in the FG group compared with the NG and HG groups at 48 h, respectively (Figures 1C,D). Flow cytometry showed that the numbers of necrotic cell were significantly increased in the FG group compared with the NG and HG groups at 48 h (Supplementary Figure 1A). Taken together, these results indicated that GF deteriorated cell viability, inflammation and apoptosis injury in MCs.

Then, we evaluated the protective effects of quercetin against GF related cytotoxicity and selected 48 h as time point for further experiments. Both moderate (20 μmol/L) and high dose (40 μmol/L) of quercetin could significantly increase MCs viability and decreased inflammation damage under FG condition (Figures 1E,F). Quercetin could reverse GF-induced cell apoptosis by reducing index of apoptosis and activity of caspase-3 (Figures 1G,H). Quercetin could reduce GF-induced cell necrosis (Supplementary Figure 1B). In general, these data suggested that GF deteriorated inflammation damage and apoptosis injury in MCs, while quercetin could alleviate this GF-triggered cytotoxicity.

### Quercetin Reversed GF-Triggered Aerobic Glycolysis in Glomerular MCs

**Figure 2:**
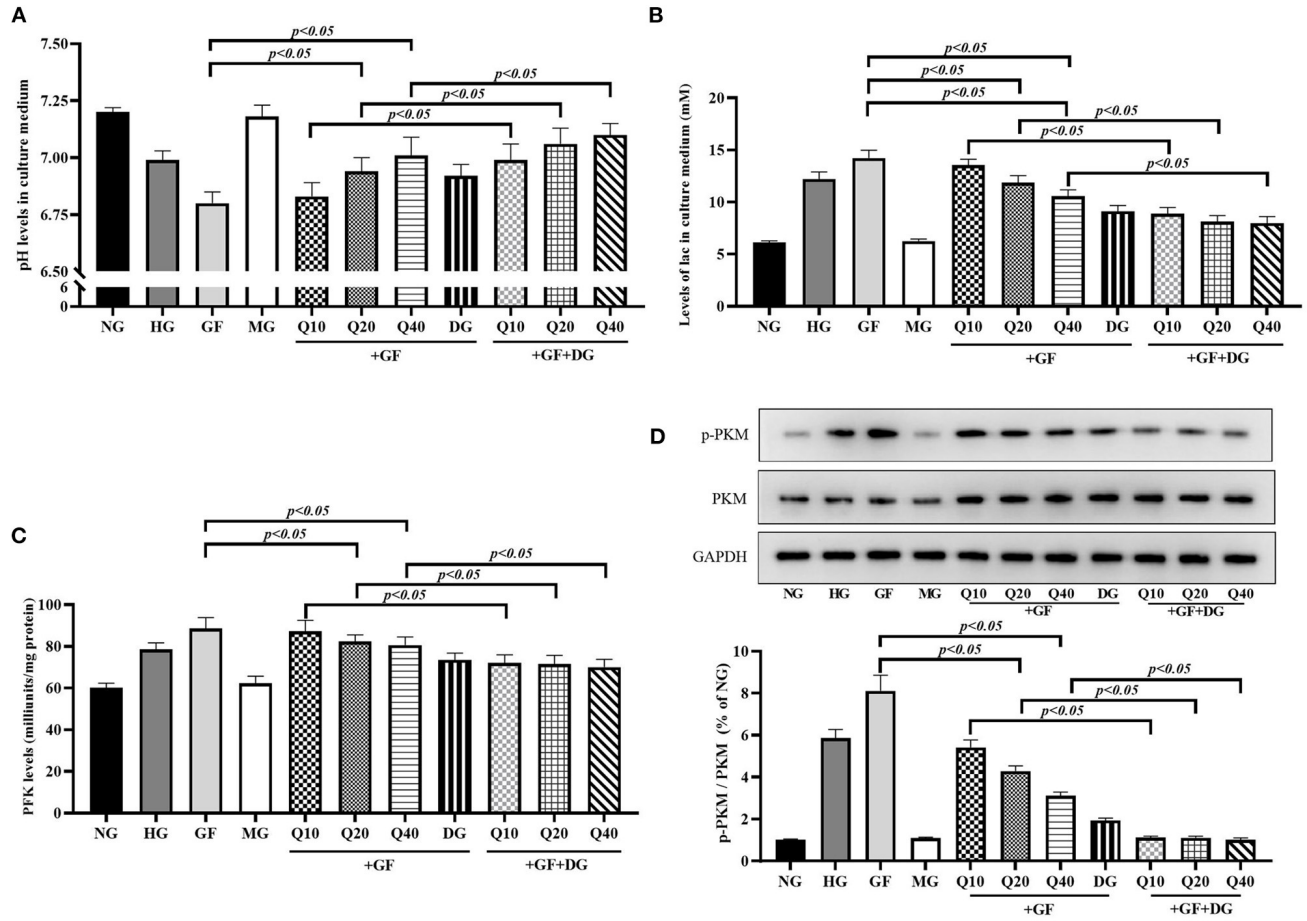
Quercetin reversed GF-triggered aerobic glycolysis in glomerular MCs. **(A)** Levels of pH in each group. **(B)** Levels of lac in each group. **(C)** Activity of PFK tested by Colorimetric Assay. **(D)** Western blot analysis showing phosphorylation levels of PKM2 in each group. As expect, these GF-triggered increase of aerobic glycolysis could be dose-dependently blocked by quercetin and glycolytic pathway inhibitor 2-deoxyglucose (2-DG). The error bar reflects the S.E.M. of at least three independent experiments.

Next, we focused on the levels of aerobic glycolysis under GF condition and the effects of quercetin against aerobic glycolysis in MCs. As presented in Figure 2, GF could remarkably induce abnormal of cellular energy metabolite levels, including reduction of pH (Figure 2A) and elevation of lac in cell culture medium (Figure 2B). Phosphofructokinase (PFK) catalyzes fructose-6-phosphate to fructose-1, 6-diphosphate and is the rate-limiting enzyme of glycolysis. The results showed that activity of PFK was enhanced under GF condition (Figure 2C). PKM2 serves as the final rate-limiting enzyme associated with cell reliance on aerobic glycolysis. We detected the level of PKM2 particularly phosphorylated PKM2 at Tyr105 (p-PKM2) in the MCs under different glucose administration. Western blot analysis showed both levels of p-PKM2/PKM2 were markedly increased (Figure 2D). As expect, these GF-triggered increase of aerobic glycolysis could be dose-dependently blocked by quercetin and glycolytic pathway inhibitor 2-deoxyglucose (2-DG). Totally, these results indicated that GF intensified aerobic glycolysis in MCs and quercetin could inhibit this intensification in a dose-dependent manner.

### Quercetin Increased Activities of FeS-Dependent Metabolic Enzymes in Condition of GF

**Figure 3:**
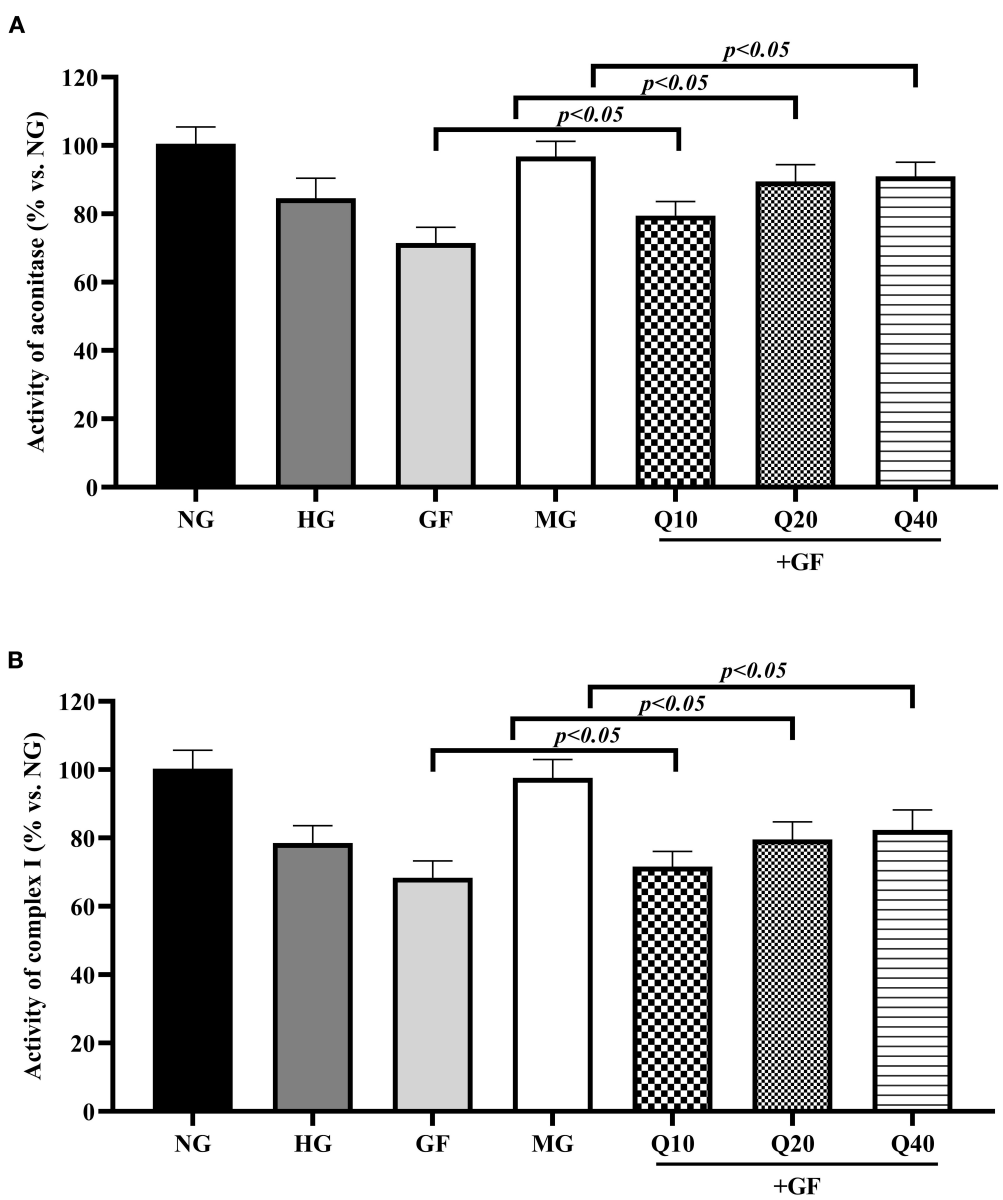
Quercetin increased activities of FeS-dependent metabolic enzymes in condition of GF. The activities of two typical FeS-dependent metabolic enzymes, aconitase **(A)**, and complex I **(B)**, were measured with colorimetric assays. The error bar reflects the S.E.M. of at least three independent experiments.

Then, we aimed to evaluate the influence of GF to oxidative phosphorylation (OXPHOS), which was an essential process for ATP generation. Cellular OXPHOS depends on a series of FeS-dependent metabolic enzymes, including aconitase and mitochondrial respiratory chain complex I. In this experiment, as shown in Figure 3, GF presented repression effects to the activity of aconitase and complex I and these suppressions were restored in the presence of quercetin. Altogether, these results suggested that quercetin prevented activities of FeS-dependent metabolic enzymes under GF injury in MCs.

### Quercetin Alleviated GF-Induced Alteration of ISCU1/2 Levels in MCs

**Figure 4:**
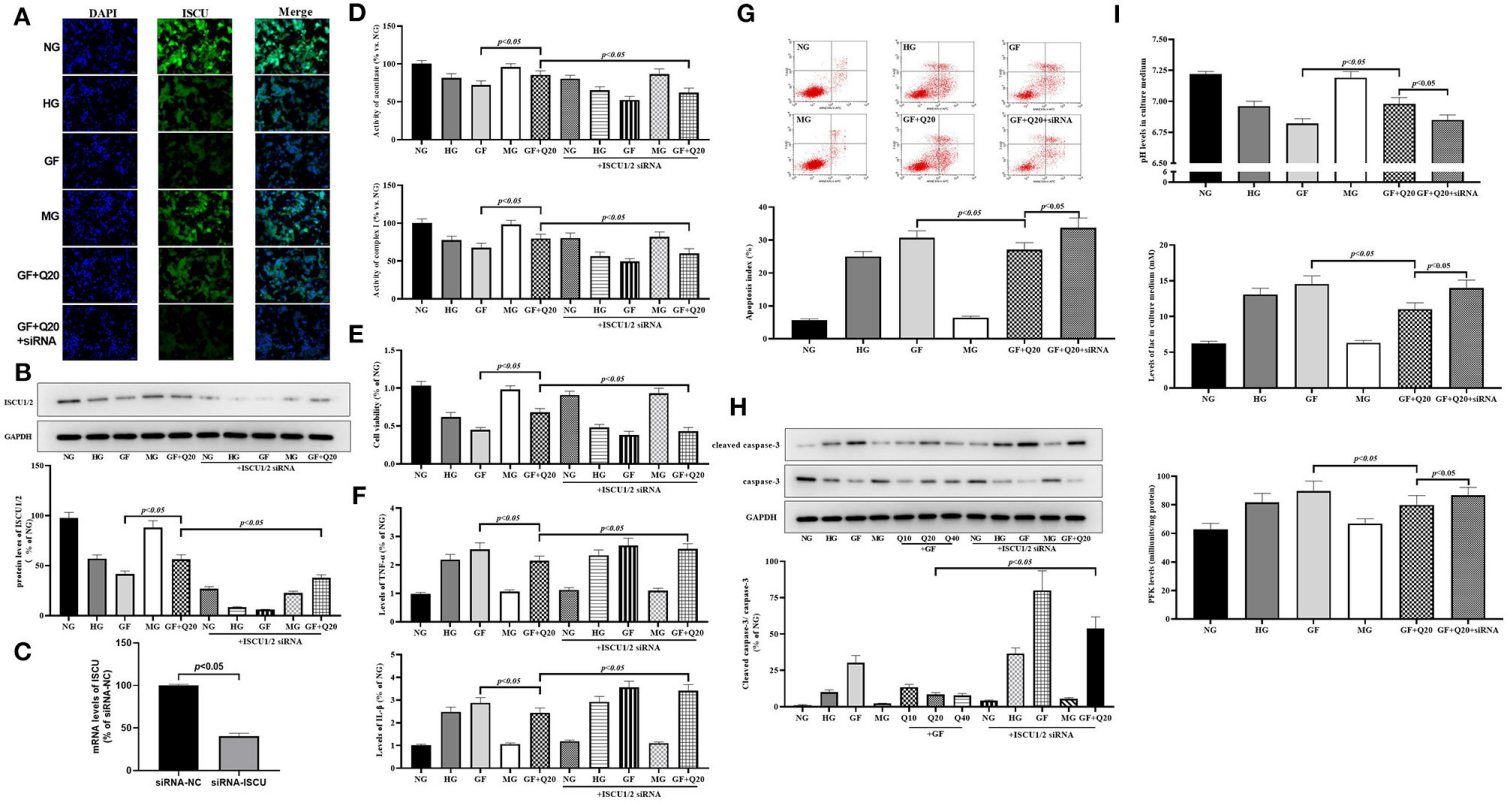
Quercetin alleviated GF-induced alteration of ISCU1/2 levels in MCs. **(A)** Representative IF staining for ISCU1/2 in MCs under different glucose. Blue indicates DAPI staining and grean means ISCU1/2 staining. Scale bar = 50 μm. **(B)** Western blot analysis showing protein levels of ISCU1/2. **(C)** Silence effect of ISCU siRNA tested by RT-qPCR. **(D)** Activities of FeS-dependent metabolic enzymes (aconitase and complex I) measured by colorimetric assays. **(E)** Viability of MCs tested by CCK8. **(F)** The effects of quercetin and ISCU1/2 siRNA on Inflammation levels of TNFα and IL-1β in MCs under different glucose. **(G,H)** The effects of quercetin and ISCU1/2 siRNA on index of apoptosis and activity of caspase-3 in MCs. **(I)** Effects of quercetin and ISCU1/2 siRNA on aerobic glycolysis activity (levels of pH, lac, and PFK). The error bar reflects the S.E.M. of at least three independent experiments.

**Figure 5:**
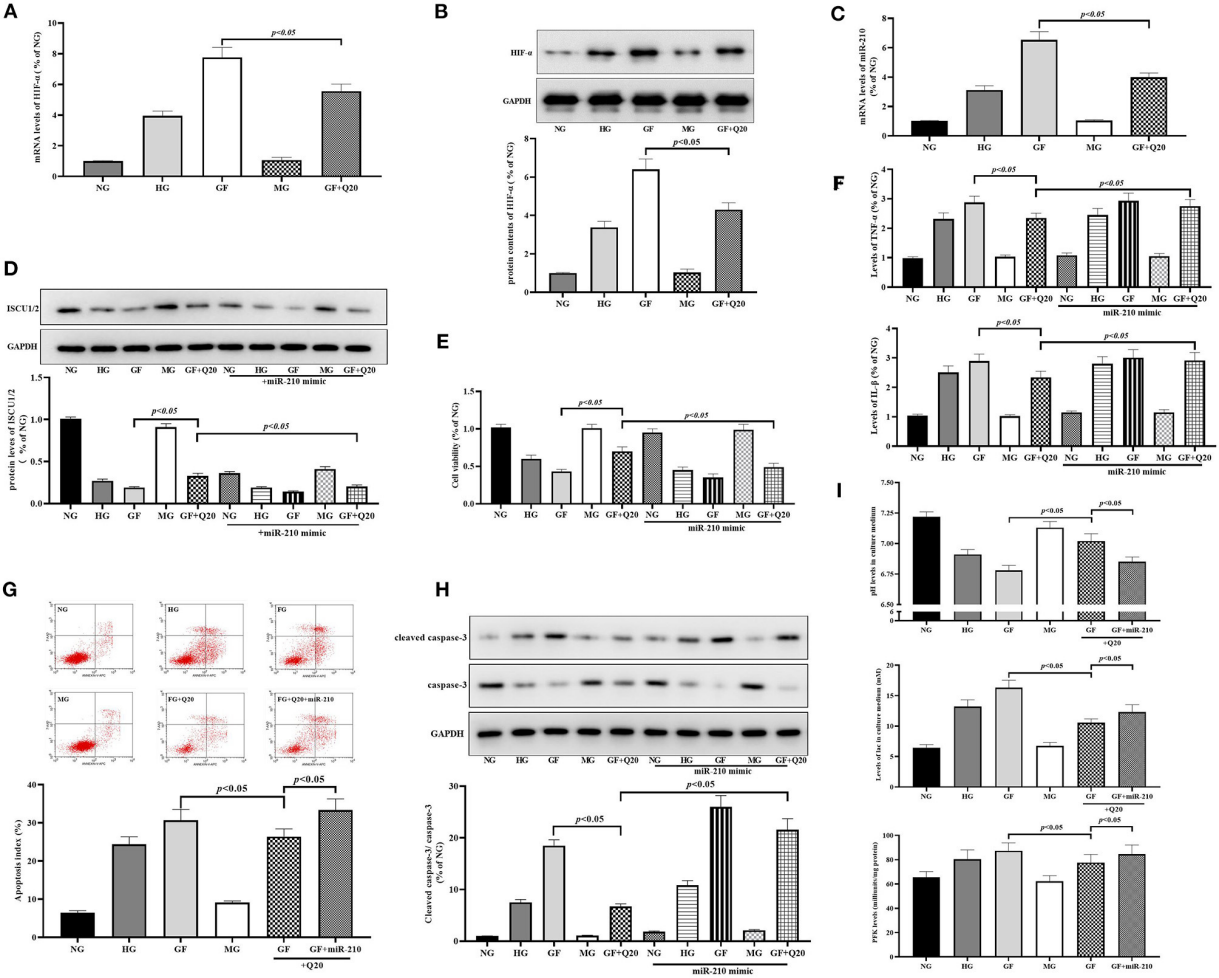
Quercetin inhibited GF-induced upregulation of HIF-1α/miR-210 levels. **(A,B)** mRNA expression and protein contents of HIF-1α measured by RT-qPCR and WB, respectively. **(C)** RT-qPCR analysis presented expression of miR-210. **(D)** Western blot analysis showing levels of ISCU1/2. **(E)** Cell viability tested by CCK8. **(F)** Levels of inflammation factors. **(G,H)** The effects of quercetin and miR-210 mimic on index of apoptosis and activity of caspase-3 in MCs. **(I)** Effects of quercetin and miR-210 mimic on aerobic glycolysis activity (levels of pH, lac, and PFK). The error bar reflects the S.E.M. of at least three independent experiments.

To elucidate the protective mechanism of quercetin against GF injury via FeS assembly, we analyzed alteration of ISCU1/2 levels, which was the up-stream regulator of FeS. First, we used immunofluorescence staining to find the reduction of ISCU1/2 under GF injury in MCs (Figure 4A). Consistent with IF results, Western blot analysis showed that GF treatment could decrease the protein levels of ISCU1/2 and quercetin could alleviate these reductions (Figure 4B). Then, we used a specific siRNA to silence ISCU1/2 (Figure 4C). Knockdown of ISCU by siRNA could weaken the effects of quercetin that maintained protein levels of ISCU1/2 (Figure 5D). ISCU siRNA could also diminish protective effects of quercetin in improving cell viability (Figure 4E), relieving inflammation injury (Figure 4F), decreasing apoptosis (Figures 4G,H) and necrosis (Supplementary Figure 1C), and reducing aerobic glycolysis switch (Figure 4I) in MCs. Therefore, these results indicated that quercetin prevented against GF injury via ISCU/FeS axis in MCs.

### Quercetin Inhibited GF-Induced Upregulation of HIF-1α/miR-210 Levels

Next, we confirmed whether HIF-1α/miR-210, as the regulator of ISCU/FeS axis, participated in the protective effects of quercetin against GF-induced injury in MCs. First, we found the mRNA expression and protein contents of HIF-1α (Figures 5A,B) were increased after GF administration, and these could be alleviated by quercetin treatment. Subsequently, RT-qPCR analysis showed that GF could trigger a sharp increase in miR-210 expression and quercetin could repress this overexpression (Figure 5C). Up-regulation of miR-210 by mimic could weaken the effects of quercetin that maintained protein levels of ISCU1/2 (Figure 4D). Then, we found miR-210 mimic could inhibit protective effects of quercetin in improving cell viability (Figure 5E), relieving inflammation injury (Figure 5F), decreasing apoptosis (Figures 5G,H) and necrosis (Supplementary Figure 1D), and decreasing aerobic glycolysis levels (Figure 5I) in MCs. Taken together, these results indicated the inhibition of quercetin against GF-induced upregulation of HIF-1α/miR-210 levels.

## Discussion

Unstable blood glucose levels have been wildly accepted to trigger more inflammation and apoptosis damages than constant high or low glucose levels in our previous study and others (4, 5, 27). Oscillating high glucose has been regard to participate in the pathogenesis of DN (3). In the present study, we proposed a cellular model using primary cultured MCs exposed to GF which partly minics glucose oscillation *in vivo* in DM patients. We observed the GF-triggered cytotoxicity in MCs, which were consisted with previous study (28). We also found that quercetin could block these damages by reducing inflammation levels and apoptotic cell numbers in MCs. Total flavones of Abelmoschus manihot has been reported as a potential therapeutic herb for the treatment of DN in our previous studies (14, 15). Quercetin, one of the bioflavonoid compounds of Abelmoschus manihot, presented protective effect against the initiation and progression of DN in diabetic mice in our previous study (21). On the basis of our previous study, the present study was performed with a mouse glomerular MCs cell line. Our finding provided further evidence in support of quercetin's kidney protection.

Aerobic glycolysis has been confirmed to engage in a series of chronic kidney pathological processes, such as inflammation and fibrosis. Ding et al. (6) found that aerobic glycolysis was increased in mouse kidney with unilateral ureter obstruction related nephropathy or TGF-beta1-treated renal interstitial fibroblasts, which indicated that aerobic glycolysis was positively correlated with kidney fibrosis process. Another study found that the aerobic glycolysis was the vital recodification of cell energy metabolism in renal tubular epithelial cell fibrosis. The increasing flux of aerobic glycolysis affected the number and function of podocytes and aggravated renal interstitial fibrosis (7). It has been demonstrated the crosstalk interaction between inflammatory cytokine TNF-α and aerobic glycolysis (29). As is well-known, both inflammation and fibrosis are the key features of DN. The results of our study showed that GF could intensify aerobic glycolysis in MCs, including reduction of pH and elevation of lac in MCs culture medium, and activation of PKM2 phosphorylation. PKM2 is the final rate-limiting enzyme associated with cell reliance on aerobic glycolysis. The recoding energy metabolism under oscillating glucose may lead a fire-new direction for research regarding GF-triggered renal injury of DN. Interestingly, our results showed quercetin could block GF-triggered increase of aerobic glycolysis. Quercetin has been reported to inhibit aerobic glycolysis levels in rat testis and some tumor cells (30, 31). Our results further proved quercetin played as an inhibitor of aerobic glycolysis in a cell model of GF-induce renal injury. The effect of quercetin to block aerobic glycolysis may increase the knowledge of quercetin in kidney protection.

To elucidate the inhibition molecular mechanism of quercetin against aerobic glycolysis, we select HIF-1α/miR210/ISCU/FeS axis. This axis has been reported as a classical regulator of aerobic glycolysis in multiple physiologic and pathologic processes (8, 32, 33). We confirmed that the expressions of HIF-1α/miR-210 were both sharply increased after GF administration and quercetin could repress these over-expressions. It has been demonstrated that oscillating glucose induced up-regulation of HIF-1α, which might play a pivotal role in the series of injuries triggered by unstable blood glucose (34). MiR-210 is a response binding element of HIF-1α. It has been reported that unstable blood glucose induced energy stress via up-regulating miR-210 in pancreatic cancer cells (35). Our finding provided the evidence that swing of glucose also induced abnormal expression of miR-210 in a mouse glomerular MCs cell line. Considering HIF-1α/miR-210 are involving in GF (11), DN (12), and Warburg effect (13), it is reasonable to believe that this axis may be a vital target in the treatment of GF-related damage and the prevention of DN. ISCU/FeS pathway is the down-stream target of HIF-1α/miR-210. FeS-dependent metabolic enzymes are essential factors in cellular OXPHOS. Our results showed that GF presented repression effect to the activity of aconitase and complex I and these suppressions were restored in the presence of quercetin in MCs. We also found knockdown of ISCU by siRNA could weaken the effects of quercetin that maintained protein levels of ISCU1/2 and activities of FeS-dependent metabolic enzymes. ISCU1/2 is the vital enzyme in the progress of FeS assembly and is the down-stream target of miR-210. Overexpression of miR-210 has been reported to disturb cellular energy metabolism and induce mitochondrial dysfunction via inhibiting ISCU1/2 in rat brain and H9c2 cardiomyocyte (36, 37).

There are some shortcomings in this study. First, we did not elucidate whether HIF-1α/miR-210/ISCU/FeS were direct or indirect targets of quercetin. Secondly, we only tested in mouse MCs but not any other cell lines related to DN, such as podocytes and endothelial cells. Thirdly, our study only experimented *in vitro* without *in vivo* experiments.

In summary, our study demonstrated that quercetin antagonized GF-induced renal injury by suppressing aerobic glycolysis via HIF-1α/miR-210/ISCU/FeS pathway in MCs cell model. Although further studies are needed, our findings may contribute to a new insight into understanding the mechanism of GF-induced renal injury and protective effects of quercetin.

## Data Availability Statement

The raw data supporting the conclusions of this article will be made available by the authors, without undue reservation.

## Author Contributions

W-lX and Y-hP: conceptualization. SL, NL, L-fY, MZ, C-yL, YZ, and QP: methodology and investigation. J-jB and X-jC: validation. Y-hP: writing—review and editing. W-lX and J-yY: supervision, project administration. All authors contributed to the article and approved the submitted version.

## Conflict of Interest

The authors declare that the research was conducted in the absence of any commercial or financial relationships that could be construed as a potential conflict of interest.

## Supplementary Material

Supplementary Figure 1 Numbers of necrotic cell detected by flow cytometry. (**A)** Numbers of necrotic cell under different glucose at 48 h. (**B)** Effects of different quercetin doses on cell necrosis. (**C)** The effects of quercetin and ISCU1/2 siRNA on necrosis in MCs. **(D)** The effects of quercetin and miR-210 mimic on necrosis in MCs. The error bar reflects the S.E.M. of at least three independent experiments. ^*^*P* < 0.05 vs. NG. ^#^*P* < 0.05 vs. HG.
