# Supplementary figures and images for: Quercetin Antagonizes Glucose Fluctuation Induced Renal Injury by Inhibiting Aerobic Glycolysis via HIF-1α/miR-210/ISCU/FeS Pathway
Source: Front Med (Lausanne). 2021 Mar 4;8:656086. doi: 10.3389/fmed.2021.656086 (PMC7969708; doi:10.3389/fmed.2021.656086)

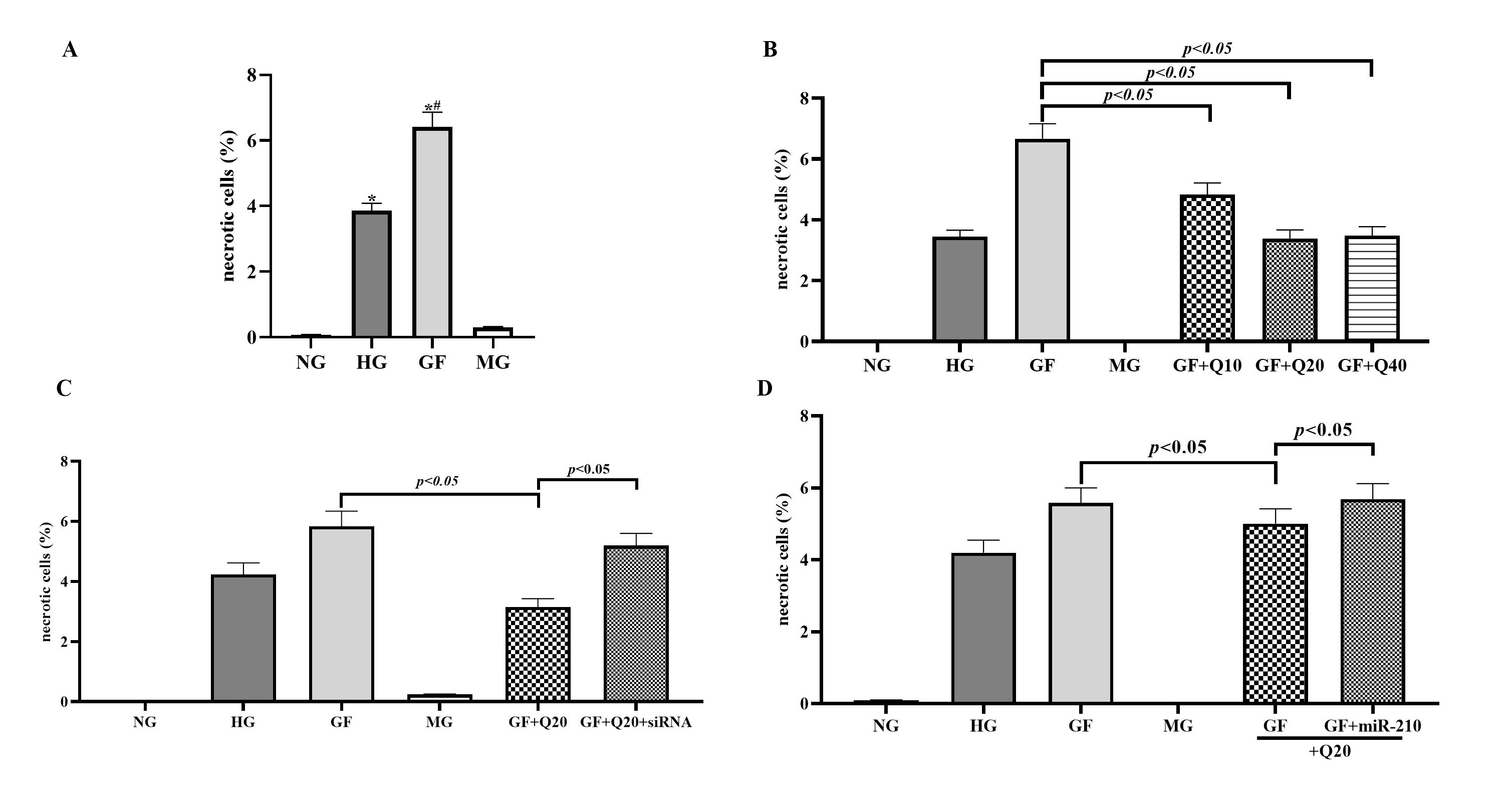

Supplement: Supplementary Figure 1 — Numbers of necrotic cell detected by flow cytometry. (A) Numbers of necrotic cell under different glucose at 48 h. (B) Effects of different quercetin doses on cell necrosis. (C) The effects of quercetin and ISCU1/2 siRNA on necrosis in MCs. (D) The effects of quercetin and miR-210 mimic on necrosis in MCs. The error bar reflects the S.E.M. of at least three independent experiments. *P < 0.05 vs. NG. #P < 0.05 vs. HG. [file Image_1.jpg]
